# Role for Endogenous BDNF in Endocannabinoid-Mediated Long-Term Depression at Neocortical Inhibitory Synapses^1^,^2^,^3^

**DOI:** 10.1523/ENEURO.0029-14.2015

**Published:** 2015-04-01

**Authors:** Liangfang Zhao, Mason Li-Wen Yeh, Eric S. Levine

**Affiliations:** Department of Neuroscience, University of Connecticut Health Center, Farmington, Connecticut 06030

**Keywords:** BDNF, CB1, endocannabinoid, inhibitory, long-term depression, trkB

## Abstract

The endogenous cannabinoid (endocannabinoid) system is an important regulator of synaptic function. Endocannabinoids acutely modulate inhibitory and excitatory transmission, and also mediate long-term depression at GABAergic and glutamatergic synapses. Typically, endocannabinoid synthesis and release is stimulated by depolarization-induced calcium influx and/or activation of phospholipase-C (PLC) signaling triggered by mGluR activation. Recently it has been shown that brain-derived neurotrophic factor (BDNF) can also induce endocannabinoid release. Although there is growing evidence for cross-talk between BDNF and endocannabinoid signaling, little is known about the functional relevance of these interactions. In the present studies, we examined BDNF−endocannabinoid interactions in regulating activity-dependent long-term depression at inhibitory synapses (iLTD). We found that theta burst stimulation (TBS) in layer 2/3 of mouse somatosensory cortical slices can induce a form of endocannabinoid-mediated iLTD that is independent of metabotropic glutamate receptor (mGluR) activation. This endocannabinoid-dependent iLTD, however, requires endogenous BDNF-trkB signaling, as it is blocked by a trk tyrosine kinase inhibitor and by a trkB receptor antagonist, and also requires activation of diacylglycerol lipase (DAG-lipase, DGL). In addition, endocannabinoid-mediated iLTD can be induced by combining a subthreshold concentration of exogenous BDNF with weak TBS stimulation that by itself is insufficient to induce iLTD. Taken together, our results suggest that TBS can induce the release of endogenous BDNF, which triggers DGL-dependent endocannabinoid mobilization and cannabinoid receptor-dependent iLTD at layer 2/3 cortical synapses.

## Abstract (precis)

Endocannabinoids and neurotrophins, particularly brain-derived neurotrophic factor (BDNF), are potent neuromodulators that play critical roles in many behavioral and physiological processes. Disruption of either BDNF or endocannabinoid signaling is associated with an overlapping set of neurologic and psychiatric diseases, and both systems are currently major targets for the development of novel therapeutics, particularly in relation to depression, anxiety, autism, and schizophrenia.

## Significance Statement

Endocannabinoids and neurotrophins, particularly brain-derived neurotrophic factor (BDNF), are potent neuromodulators that play critical roles in many behavioral and physiological processes. Disruption of either BDNF or endocannabinoid signaling is associated with an overlapping set of neurologic and psychiatric diseases, and both systems are currently major targets for the development of novel therapeutics, particularly in relation to depression, anxiety, autism, and schizophrenia. Little is known, however, about the interactions between these systems. In the present studies, we find that endogenous BDNF is released in an activity-dependent manner to trigger endocannabinoid-mediated depression of inhibitory synapses. A mechanistic understanding of BDNF-endocannabinoid interactions regulating synaptic plasticity may provide clues to underlying pathologies of neurologic and psychiatric disorders and suggest novel strategies for therapeutic intervention.

## Introduction

Endogenous cannabinoids (endocannabinoids, eCBs) are important regulators of synaptic function in the nervous system. In particular, endocannabinoids acutely modulate inhibitory and excitatory transmission throughout the forebrain, and mediate several forms of short-term plasticity at GABAergic and glutamatergic synapses, including depolarization-induced suppression of inhibition (DSI) and excitation (DSE). During DSI or DSE, endocannabinoids are released from postsynaptic sites in response to depolarization-induced calcium influx and act retrogradely via presynaptic type-1 cannabinoid receptors (CB1Rs) to suppress transmitter release. Endocannabinoids also mediate specific types of long-term depression at excitatory and inhibitory synapses (for review, see Chevaleyre et al., 2006; Mackie, 2008; Cachope, 2012). These forms of plasticity are dependent on CB1Rs, which are highly expressed throughout the forebrain, and are activated by both arachidonoylethanolamide (anandamide) and 2-arachidonoylglycerol (2-AG), the two best-characterized endogenous ligands of CB1R. It has been shown that the on-demand synthesis and release of endocannabinoids in the hippocampus, neocortex, cerebellum, and other areas can be stimulated by depolarization-induced calcium influx, as well as by activation of phospholipase-C_β_ (PLCβ) triggered by G_q_-protein coupled receptors, particularly metabotropic glutamate receptors (mGluRs) (for review, see Hashimotodani et al., 2007; Castillo et al., 2012).

Recently it has been shown that brain-derived neurotrophic factor (BDNF) can also induce endocannabinoid release, part of a growing body of evidence for interactions between endocannabinoid signaling and BDNF. For example, there is evidence of eCB–BDNF interactions in visual cortex (Huang et al., 2008), hippocampus (Khaspekov et al., 2004; Roloff et al., 2010), and cerebellum (Maison et al., 2009). In support of this, there is strong colocalization of trkB receptors and CB1R throughout the forebrain. Within the neocortex, trkB is predominantly localized to layers 2/3 and 5 (Cabelli et al., 1996; Fryer et al., 1996; Miller and Pitts, 2000), and the highest levels of CB1R in the neocortex are also found in layer 2/3 (Matsuda et al., 1993; Tsou et al., 1998; Marsicano and Lutz, 1999; Egertová et al., 2003). Recent studies in the neocortex have shown that endocannabinoid synthesis and release can be rapidly mobilized by BDNF−trkB signaling. In neocortical layer 2/3, acute application of BDNF rapidly suppresses GABAergic transmission via release of endocannabinoids from the postsynaptic pyramidal cell, which act in a retrograde manner to suppress presynaptic transmitter release (Lemtiri-Chlieh and Levine, 2010). This effect of BDNF is initiated by postsynaptic trkB signaling, requires downstream PLC signaling, and is independent of mGluR activation (Zhao and Levine, 2014).

Endogenous BDNF has been shown to play a critical role in LTP at excitatory synapses, but its role at inhibitory synapses and its potential role in endocannabinoid-mediated synaptic plasticity are less clear. Because exogenous BDNF can trigger endocannabinoid mobilization that suppresses GABA release at cortical synapses, we explored whether endogenous BDNF plays a role in long-term depression at cortical inhibitory synapses. In particular, we hypothesized that stimulation-induced release of BDNF, acting through trkB and downstream diacylglycerol lipase (DGL) signaling, can trigger endocannabinoid release to cause long-term depression at cortical inhibitory synapses.

## Materials and Methods

### Animal handling and slice preparation

All animal procedures are performed according to the regulation of Authors University’s Institutional Animal Care and Use Committee. Postnatal day 15-27 Swiss CD-1 mice of either sex (Charles River) were anesthetized by 3.5% isoflurane inhalation, followed by decapitation. Whole brains were removed and immersed in ice-cold slicing solution containing (in mM): 110 choline chloride, 2.5 KCl, 1.25 NaH_2_PO_4_·H_2_O, 25 NaHCO_3_, 0.5 CaCl_2_, 7 MgCl_2_·6H_2_O, 25 dextrose, 11.6 sodium ascorbate, 3.1 sodium pyruvate, equilibrated with 95% O_2_-5% CO_2_ (pH 7.3, 310 ± 5 mOsm/kg). Transverse slices (350 µm) containing somatosensory cortex were cut with a Dosaka EM DTK-1000 vibratome and transferred to an incubating chamber. Slices were then incubated for 15 min at 33-35 °C in carboxygenated incubating solution containing (in mM): 125 NaCl, 2.5 KCl, 1.25 NaH_2_PO_4_, 25 NaHCO_3_, 0.5 CaCl_2_, 3.5 MgCl_2_·6H_2_O, 4 sodium lactate, 2 sodium pyruvate, 25 dextrose, and 0.4 ascorbic acid (pH 7.3, 310 ± 5 mOsm/kg) before being transferred to room temperature. Slices were then individually transferred to a recording chamber (at room temperature) and fixed to the stage of an Olympus BX51WI upright microscope fitted with a 40× water-immersion objective lens (0.8 NA). The recording chamber was continuously perfused at 1-1.5 ml/min with carboxygenated artificial CSF (aCSF) consisting of (in mM): 125 NaCl, 2.5 KCl, 1.25 NaH_2_PO_4_, 25 NaHCO_3_, 2 CaCl_2_, 2 MgCl_2_·6H_2_O, 25 dextrose (pH 7.3, 305 ± 5 mOsm/kg).

### Electrophysiology

Whole-cell recordings were obtained from layer 2/3 or layer 5 somatosensory cortex pyramidal neurons. Pyramidal neurons were identified by their morphology under infrared differential interference contrast video microscopy. Patch electrodes (2-3 MΩ) were pulled from borosilicate glass capillaries using a Flaming/Brown P-97 micropipette puller (Sutter Instrument). Pipette internal solution for recording IPSCs contained (in mM): 130 CsCl, 10 HEPES, 1 EGTA, 0.1 CaCl_2_, 1.5 MgCl_2_, 4 Na_2_-ATP, 0.3 Na-GTP, 10 di-tris-phosphocreatine and 5 QX-314 (pH 7.3, 290 ± 5 mOsm/kg). The chloride equilibrium potential (*E*_Cl_) using this internal solution was close to 0 mV; thus, IPSCs were recorded as inward currents. Ionotropic glutamate receptor antagonists 6, 7-dinitroquinoxaline-2, 3-dione (DNQX; 10 µM) and 3-[(*R*)-2-carboxypiperazin-4-yl]-propyl-1-phosphonic acid (CPP; 3 µM) were used to isolate inhibitory activity. A bipolar tungsten electrode (1 MΩ; WPI) was positioned 70-150 µm lateral to the patched pyramidal neuron to elicit electrically-evoked IPSCs (eIPSCs). Extracellular stimuli consisted of individual square-wave current pulses (170 µs; 4-30 µA) and were delivered every 20 seconds through a stimulus isolator (ISO-Flex; A.M.P.I). Every third stimulation was delivered using a paired-pulse protocol (75 ms interpulse interval). Stimulation strength was set to a level that evoked 30-70% of maximal response for each individual cell. For the pipette internal solution for recording EPSCs, 130 CsCl was replaced with 118.7 CH_3_O_3_SCs and 6.3 CsCl, thus the *E*_Cl_ using this internal solution was close to −70 mV.

Cells were voltage clamped at −70 mV during recording. All electrical events were filtered at 2.9 kHz and digitized at >6 kHz using a HEKA EPC9 amplifier and ITC-16 digitizer (HEKA Elektronik). Series resistance (*R*_s_) was compensated up to 40% at 100 µs lag. Input resistance (*R*_i_) was monitored with 10 mV (50 ms) hyperpolarizing voltage steps at the end of each sweep. Cells were rejected from analysis if *R*_i_ fell below 50 MΩ or the holding current dropped by 150 pA during the course of an experiment.

### Chemicals

Unless otherwise stated, all drugs were from Tocris Biosciences and were delivered by bath perfusion. Drugs were first prepared as concentrated stock solution in solvents and stored at −20 °C. The stock solutions of DNQX, AM251, ANA-12, K252a, SR 141716A, and tetrahydrolipstatin (THL) were dissolved in 100% dimethyl sulfoxide (DMSO). The final concentration of DMSO did not exceed 0.1%, which by itself had no effect on evoked inhibitory synaptic currents (Zhao and Levine, 2014) and had no effect on evoked excitatory currents (102.5 ± 0.3% of baseline, *n* = 3). CPP, E4CPG, and BDNF (PeproTech) were dissolved in 18 MΩ water. Drug stock solutions were diluted into aCSF on the day of recording to the final concentrations. Bovine serum albumin (BSA; Sigma-Aldrich) was added to the BDNF solution at a concentration of 0.1 g/l to reduce nonspecific binding. BSA by itself had no effect on evoked IPSC amplitude (112.7 ± 13.2% of baseline, *n* = 3). Preincubation time for all drugs was 15-30 min prior to experiment.

### Data analysis

Off-line analysis was carried out using Clampfit 10 (Molecular Devices) and Prism 6 (GraphPad Software). Statistical comparisons were made between average amplitudes of baseline responses and 27-35 min postinduction using two-tailed Student’s paired *t* test unless otherwise stated. *p* < 0.05 was taken as a statistically significant effect. In individual examples, sweeps of evoked responses were averaged traces of four consecutive evoked IPSCs around corresponding time windows. Group data are reported as mean ± SEM.

## Results

### Strong theta frequency burst stimulation induces eCB-dependent iLTD

**Figure 1:**
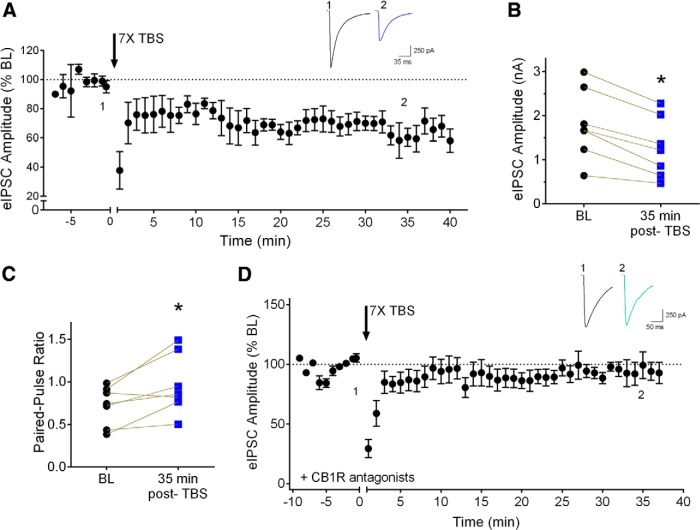
TBS in layer 2/3 induces CB1R-dependent iLTD. ***A***, Group time course of the effect of seven trains of TBS (7× TBS) on normalized peak eIPSC amplitudes (*n* = 7). Arrow indicates TBS stimulation (this and all following figures). Inset, Averaged sample sweeps during baseline (1) and 35 min after TBS (2) from a representative experiment. ***B***, Data from individual experiments showing eIPSC amplitude during baseline (black circles, BL) and 35 min after TBS (blue squares). *p < 0.05 compared to baseline. ***C***, Paired-pulse ratio data from the same cells as in ***B*** during baseline (black circles, BL) and 35 min after TBS (blue squares). *p < 0.05 compared to baseline. ***D***, Group time course showing lack of effect of 7× TBS on peak eIPSC amplitude in the presence of the CB1R antagonists AM251 (5 μM) or SR 141716A (10 μM, *n* = 9 in total). Inset, Averaged sample sweeps during baseline (1) and 35 min after TBS (2) from a representative experiment.

**Figure 2:**
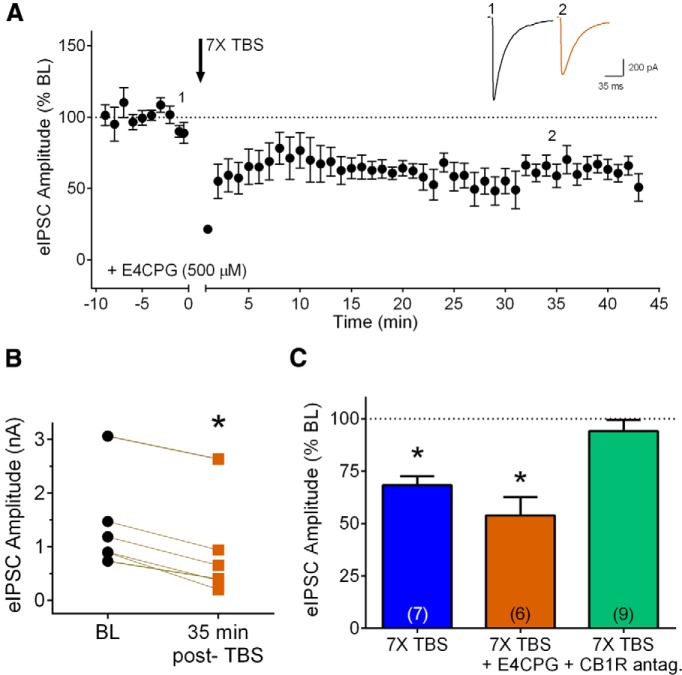
iLTD at layer 2/3 inhibitory synapses is independent of mGluR signaling. ***A***, Group time course of the effect of 7× TBS on peak eIPSC amplitude in the presence of the group I/II mGluR antagonist E4CPG (500 μM; *n* = 6). Inset, Averaged sample sweeps during baseline (1) and 35 min after TBS (2) from a representative experiment. ***B***, Data from individual experiments showing eIPSC amplitude baseline (black circles, BL) and 35 min after TBS in the presence of E4CPG (orange squares). *p < 0.05 compared to baseline. ***C***, Compiled group data for normalized eIPSC amplitude 35 min after 7× TBS in vehicle (blue bar), E4CPG (orange bar), or CB1R antagonists (CB1R antag., green bar). Numbers on bars indicate number of cells in each group (this and all following figures).

Evoked IPSCs were recorded from layer 2/3 pyramidal neurons in response to intralaminar stimulation (0.05 Hz). In the absence of high frequency repetitive stimulation, these responses were stable over time (101.7 ± 2.6% of baseline, *n* = 4, after 40 min of stimulation). Using theta-frequency burst stimulation (TBS), we examined long-term depression at inhibitory synapses (iLTD) at inhibitory synapses onto layer 2/3 cortical pyramidal neurons. Stimulation consisted of seven trains of TBS (7× TBS) delivered with a 5 s intertrain interval. Each TBS train contained 10 bursts (200 ms interburst interval), each burst consisted of five stimuli at 100 Hz. This protocol induced a stable long-lasting suppression of inhibitory transmission (Fig. 1*A*). As shown in Figure 1*B*, eIPSC amplitude was reduced in all seven cells tested at 35 min post-TBS (68.32 ± 4.2% of baseline, *p* = 0.0005, *n* = 7; see also Fig. 2*C*). In two cells that lasted 60 min post-TBS, eIPSC amplitude was 65.11 ± 1.4% of baseline, suggesting that suppression reached a stable plateau by 35 min postinduction.

This TBS-induced iLTD was independent of ionotropic glutamate receptor signaling because it was evoked in the presence of NMDA and non-NMDA receptor antagonists (see Materials and Methods, above). To confirm that these receptors were effectively blocked during the repetitive stimulation used to induce iLTD, we recorded evoked EPSCs in layer 5 pyramidal neurons. Intralaminar stimulation evoked a dual component EPSC that was almost completely abolished in the presence of CPP and DNQX (12.6 ± 1.6% of predrug baseline, *n* = 4). Following 7× TBS stimulation, there was no change in the amplitude of the residual evoked response (1 min post-TBS; 94.5 ± 4.7% of pre-TBS baseline, *n* = 4), indicating effective antagonism of these receptors during repetitive stimulation.

We next examined whether this iLTD requires CB1R signaling. Because CB1Rs are predominantly expressed on presynaptic terminals, we hypothesized that there would be a change in the paired-pulse ratio before and after iLTD induction. We found that the paired-pulse ratio was significantly increased after iLTD induction (137.3 ± 13.3% of baseline, *p* = 0.025, *n* = 7; Fig. 1*C*). We then examined the effect of 7× TBS during bath application of the selective CB1R antagonist AM251 (5 µM) or SR141716A (5 µM). Identical results were obtained using these structurally similar drugs, so the results were combined. As shown in the group data in Figures 1*D*and 2*C*, 7× TBS in the presence of either AM251 or SR141716A failed to induce iLTD at these synapses (94.1 ± 5.4% of baseline, *p* = 0.3201, *n* = 9; baseline, 1.17 ± 0.1 nA; 35 min-post, 1.10 ± 0.1 nA). Application of CB1R antagonists had no effect on basal eIPSC amplitude of layer 2/3 pyramidal neurons under similar conditions (Trettel and Levine, 2002). These results suggest that TBS induces eCB-dependent iLTD at inhibitory synapses onto layer 2/3 pyramidal neurons.

### iLTD in layer 2/3 of somatosensory cortex is independent of mGluR signaling

Because many forms of eCB-mediated LTD require mGluR signaling (for review, see Chevaleyre et al., 2006; Kano et al., 2009), we examined the effects of 7× TBS during bath application of the group I/group II mGluR antagonist E4CPG (500 µM). Interestingly, blocking mGluR signaling did not prevent 7× TBS from inducing iLTD at layer 2/3 inhibitory synapses (Fig. 2*A*). At 35 min postinduction, eIPSC amplitude was significantly reduced compared to baseline (Fig. 2*B*,*C*, 53.88 ± 8.7% of baseline, *p* = 0.0001, *n* = 6). The amount of suppression was comparable to that caused by 7X TBS alone (Fig. 2*C*, *p* = 0.1447, unpaired *t* test). Previously it has been shown that this concentration of E4CPG is sufficient to block the effect of an mGluR agonist on synaptic transmission in layer 2/3 (Zhao and Levine, 2014). As an additional control to confirm the efficacy of E4CPG during repetitive stimulation, we examined its effects on LTD at layer 5 excitatory synapses. The same stimulation protocol (7× TBS) induced significant LTD at these synapses (60.1 ± 4.5% of baseline, *p* = 0.0003, *n* = 7), and this suppression was blocked by E4CPG (*n* = 6, unpaired *t* test, *p* = 0.0312).

### iLTD in layer 2/3 of somatosensory cortex requires endogenous BDNF and DGL signaling

**Figure 3:**
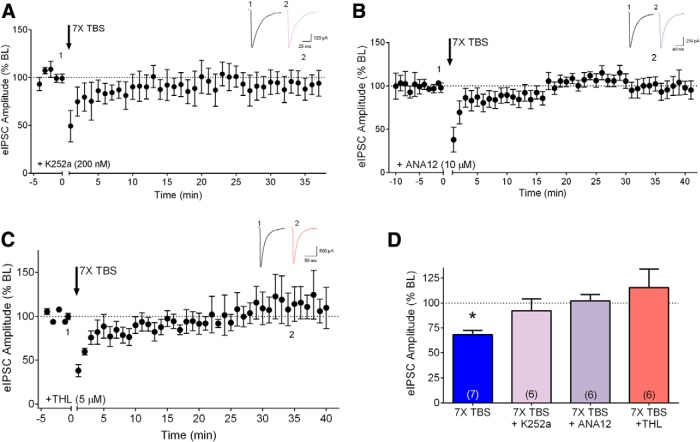
eCB-mediated iLTD requires endogenous BDNF signaling as well as DGL. ***A***, Group time course showing the lack of effect of seven trains of TBS (7× TBS) on peak eIPSC amplitude in the presence of the tyrosine kinase inhibitor K252a (200 nM, *n* = 6). ***B***, Group time course showing the lack of iLTD in response to 7× TBS in the presence of the trkB receptor antagonist ANA12 (10 μM, *n* = 6). Inset, Averaged sample sweeps during baseline (1) and 35 min after TBS (2) from a representative experiment. ***C***, Group time course showing the lack of iLTD in response to 7× TBS in the presence of DGL inhibitor THL (5 μM, *n* = 6). ***D***, Compiled group data for normalized eIPSC amplitude at 35 min postinduction in the presence of K252a (light pink bar), ANA-12 (light purple bar), or THL (coral bar). The effect of 7× TBS in vehicle (blue bar) was taken from Figure 2*C* for comparison. *p < 0.05 compared to baseline.

In addition to the mGluR/PLC_β_ signaling pathway that is known to induce eCB mobilization and release, recent studies have found that BDNF/trkB signaling, via downstream PLCγ activation, can induce eCB release at inhibitory synapses in layer 2/3 (Lemtiri-Chlieh and Levine, 2010; Zhao and Levine, 2014). Furthermore, TBS can induce release of endogenous BDNF (Li et al., 2010; for review, see Panja and Bramham, 2014). We therefore examined whether endogenous BDNF plays a role in eCB-mediated iLTD in layer 2/3. The first set of experiments used K252a (200 nM), a relatively specific inhibitor of autophosphorylation of trk tyrosine kinase receptors at this concentration (Hashimoto, 1988; Berg et al., 1992; Knüsel and Hefti, 1992; Nye et al., 1992). As shown in Figure 3, *A* and *D*, iLTD produced by 7× TBS was prevented by bath application of K252a (92.17 ± 11.8% of baseline, *p* = 0.6175, *n* = 6; baseline, 0.73 ± 0.1 nA; 35 min-post, 0.68 ± 0.1 nA). K252a alone does not have any significant effect on eIPSC amplitude in layer 2/3 slices (Lemtiri-Chlieh and Levine, 2010). A role for BDNF-trkB signaling was confirmed with the selective trkB receptor antagonist, ANA-12 (10 µM) (Cazorla et al., 2011; Alder et al., 2013; Santafé et al., 2014). As shown in Figure 3, *B* and *D*, bath application of ANA-12 completely blocked iLTD (102.2 ± 6.2% of baseline, *p* = 0.5711, *n* = 6; baseline, 1.18 ± 0.2 nA; 35 min-post, 1.24 ± 0.3 nA). ANA12 alone did not have any effect on inhibitory synaptic transmission (95.3 ± 2.5% of baseline after 10 min application, *n* = 2).

It has been previously shown that BDNF induces eCB mobilization via DGL signaling (Lemtiri-Chlieh and Levine, 2010). We thus examined whether DGL plays a role in iLTD in layer 2/3. As seen in Figure 3, *C* and *D*, in the presence of the DGL inhibitor THL (5 μM), iLTD was completely abolished (115.6 ± 18.4% of baseline, *p* = 0.5057, *n* = 6; baseline, 1.13 ± 0.2 nA; 35 min-post, 1.24 ± 0.2 nA). Taken together, these findings suggest that endogenous BDNF−trkB signaling and downstream activation of DGL, but not mGluR activation, is necessary for eCB-mediated iLTD at inhibitory synapses in layer 2/3 pyramidal neurons.

### Exogenous BDNF facilitates iLTD induction

**Figure 4:**
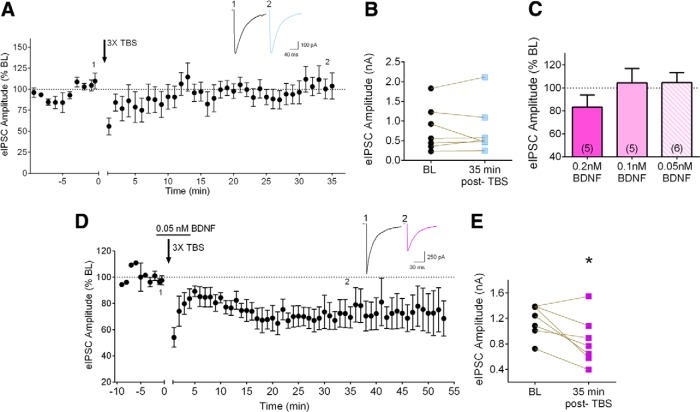
Low concentration of BDNF synergizes with subthreshold TBS to induce iLTD. ***A***, Group time course of the effect of three trains of TBS (3× TBS) on normalized peak eIPSC amplitude. Inset, Averaged sample sweeps during baseline (1) and 35 min after TBS (2) from a representative experiment (*n* = 7). ***B***, Data from individual experiments showing eIPSC amplitude baseline (black circles, BL) and 35 min after 3× TBS (light blue squares). ***C***, Dose response of the acute effect of BDNF (5 min) on eIPSC amplitude. Left bar, 5 ng/ml (0.2 nM) BDNF; middle bar, 2.5 ng/ml (0.1 nM) BDNF; right bar, 1.25 ng/ml (0.05 nM) BDNF. ***D***, Group time course of peak eIPSC amplitude under the combination of 3× TBS and 5 min-0.05 nM BDNF. Horizontal bar above trace indicates BDNF application. ***E***, Data from individual experiments showing eIPSC amplitude during baseline (black circles, BL) and 35 min after 3× TBS and 5 min-0.05 nM BDNF (purple squares). Time window is relative to TBS (for this and Fig. 5). *p < 0.05 compared to baseline.

**Figure 5:**
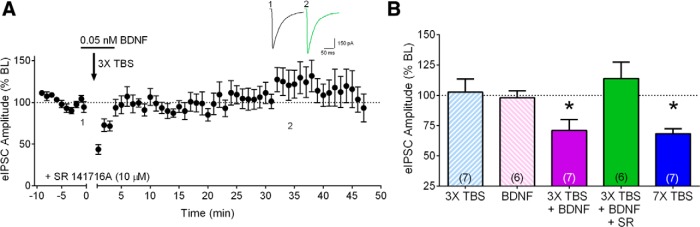
Synergized iLTD requires CB1R signaling. ***A***, Group time course illustrating the lack of effect of 3× TBS-5 min BDNF combination on eIPSC amplitude in the presence of CB1R antagonist SR 141716A (SR, 10 μM, *n* = 6.) Inset, Averaged sample sweeps during baseline (1) and 35 min after TBS (2) from a representative experiment. ***B***, Compiled group data for normalized eIPSC amplitude at 35 min post various induction conditions. Hatched light blue bar, 3× TBS. Hatched pink bar, 0.05 nM BDNF for 5 min. Purple bar, Combination of 5 min-0.05 nM BDNF and 3× TBS. Green bar, Combination of 5 min-0.05 nM BDNF and 3× TBS, in the presence of SR. Blue bar, 7tr-TBS (taken from Fig. 2*C* for comparison).

We next examined whether exogenous BDNF could facilitate iLTD induction by a subthreshold stimulus train. In contrast to the stable suppression caused by 7× TBS, three trains of TBS (3× TBS) did not induce significant iLTD, as shown in the group data in Figures 4, *A* and *B*, and 5*B* (102.6 ± 10.9% of baseline, *p* = 0.8943, *n* = 7; baseline, 0.79 ± 0.2 nA, 35 min-post, 0.78 ± 0.2 nA). In fact, only one of seven cells showed suppression with 3× TBS (Fig. 4*B*). We then examined the effects of BDNF alone on inhibitory synaptic transmission. It has previously been shown that BDNF at a concentration of 20 ng/ml (0.8 nM) significantly reduces inhibitory synaptic transmission (Lemtiri-Chlieh and Levine, 2010; Zhao and Levine, 2014). To determine a concentration of BDNF that does not by itself influence synaptic transmission, we examined the effect of 5 min bath-applied BDNF on eIPSC amplitude. Bath application of 0.2 nM (5 ng/ml) BDNF for 5 min caused a suppression of eIPSC amplitude in four of five cells, although this did not reach statistical significance (83.24 ± 10.5% of baseline, *p* = 0.2644, *n* = 5). At a concentration of 0.1 nM (2.5 ng/ml), BDNF did not cause suppression (104.4 ± 12.5% of baseline, *n* = 5), nor did BDNF at 0.05 nM (104.5 ± 8.8% of baseline; *p* = 0.7086, *n* = 6), as shown in Figure 4*C*. In addition, 0.05 nM BDNF for 5 min had no effect on eIPSC amplitude measured 35 min post-BDNF application (97.92 ± 5.8% of baseline, *p* = 0.8288, *n* = 6), indicating that brief application of 0.05 nM BDNF does not induce iLTD. Interestingly, however, the combination of 3× TBS with 0.05 nM BDNF (applied for 5 min around the time of TBS) produced significant iLTD (Figs. 4*D*,*E*, 5*B*, 70.95 ± 9.1% of baseline, *p* = 0.0305, *n* = 7), with six of seven cells showing suppression. The average amount of suppression produced by BDNF + 3× TBS was not significantly different than that produced by 7× TBS alone (Fig. 5*B*, *p* = 0.7978, unpaired *t* test). These data suggest that a subthreshold concentration of BDNF can synergize with weak TBS stimulation to induce iLTD at layer 2/3 inhibitory synapses.

Finally, we examined the downstream mechanisms underlying BDNF + 3× TBS-induced iLTD. The suppression induced by BDNF + 3× TBS was completely abolished in the presence of the CB1R antagonist SR 141716A (10 µM), as shown in Figure 5 (113.9 ± 13.4% of baseline, *p* = 0.3894, *n* = 6; baseline, 1.14 ± 0.2 nA; 35 min-post, 1.35 ± 0.4 nA). These results indicate that iLTD induced by 3× TBS + BDNF required CB1R activation, similar to 7× TBS-induced iLTD.

## Discussion

In the present study, we investigated the interaction between BDNF and eCB signaling in iLTD. We found that seven trains of TBS induce stable eCB-mediated iLTD at cortical layer 2/3 pyramidal neurons, as inhibiting CB1R activation diminished this suppression. Surprisingly, this form of iLTD is independent of mGluR activation, in contrast to the most prevalent forms of eCB-mediated LTD (Maejima et al., 2001; Chevaleyre and Castillo, 2003; Bender et al., 2006; Lafourcade et al., 2007; Chiu et al., 2010; Jiang et al., 2010). In contrast, BDNF, which has also been shown to mobilize eCBs (Lemtiri-Chlieh and Levine, 2010; Zhao and Levine, 2014) is required for this iLTD, as inhibiting trkB tyrosine kinase activity or blocking trkB receptor activation prevented TBS-induced iLTD. Consistent with previous findings that DGL is required for BDNF-induced eCB mobilization (Lemtiri-Chlieh and Levine, 2010), the present results indicate that endogenous BDNF regulates eCB-mediated iLTD via DGL, as the DGL inhibitor THL diminished iLTD. Furthermore, we also found that a low concentration of exogenous BDNF, which has no effect on its own, synergizes with three trains of TBS to induce CB1R-dependent iLTD.

The seven-train TBS protocol induced a form of iLTD that requires BDNF/trkB signaling. The source of endogenous BDNF in this form of iLTD is not clear, but potential sources would include release from presynaptic terminals or dendritic release from neighboring neurons. Previous studies have identified BDNF-containing vesicles in presynaptic glutamatergic terminals (Hartmann et al., 2001; Brigadski et al., 2005; Dean et al., 2012; Dieni et al., 2012) and stimulation has been shown to induce the release of endogenous BDNF from glutamatergic terminals (Aloyz et al., 1999; Hartmann et al., 2001; Kohara et al., 2001; Dean et al., 2012). BDNF release from inhibitory terminals is not likely as BDNF mRNA is not expressed in interneurons (Ernfors et al., 1990; Gorba and Wahle, 1999).

BDNF has also been localized to vesicles in postsynaptic dendrites (Aoki et al., 2000; Avwenagha et al., 2006) and has been shown to be released from dendrites (Hartmann et al., 2001; Kuczewski et al., 2008; Matsuda et al., 2009; Dean et al., 2012). Dendritic release of BDNF can be triggered by the following: (1) Ca^2+^ influx through ionotropic glutamate receptors or voltage-gated channels (Hartmann et al., 2001); (2) activation of Group I mGluR receptors, which subsequently triggers IP3-mediated Ca^2+^ release from intracellular calcium store (Canossa et al., 2001); and (3) Ca^2+^-dependent Ca^2+^ release from intracellular stores by ryanodine receptors (Balkowiec and Katz, 2002). However, in the present studies, iLTD was induced in the presence of both ionotropic and metabotropic glutamate receptor antagonists, and postsynaptic cells were voltage-clamped at a hyperpolarized membrane potential, arguing against postsynaptic release via these pathways. Astrocytes can also release BDNF, however this also requires mGluR signaling (Jean et al., 2008). Thus presynaptic release is the most likely source of the endogenous BDNF mediating the observed iLTD.

The location of the trkB receptors that are activated by endogenously-released BDNF in the present study is most likely on dendrites of postsynaptic pyramidal neurons in layer 2/3. There are high levels of trkB expression in postsynaptic dendrites in hippocampus and in cortex, particularly in layer 2/3 (Drake et al., 1999; Aoki et al., 2000), although there is also evidence for trkB expression at presynaptic sites (Xu et al., 2000; Kohara et al., 2001; Inagaki et al., 2008). Importantly, however, postsynaptic trkB receptor activation has been shown to be specifically required for exogenous BDNF-induced eCB release (Lemtiri-Chlieh and Levine, 2010). Postsynaptic calcium has also been shown to be required for BDNF-induced eCB release (Lemtiri-Chlieh and Levine, 2010); thus, release of calcium from intracellular stores may also be involved in the eCB-mediated LTD reported in the present studies.

Activation of postsynaptic trkB receptors induces the synthesis and release of eCBs that act retrogradely on presynaptic CB1Rs to trigger iLTD. The endogenous CB1R ligand that mediates iLTD in the present study is not completely clear, but is likely to be 2-AG. Activation of PLCγ and PLCβ, downstream effectors of BDNF/trkB and mGluR, respectively, leads to cleavage of phosphatidylinositol 4,5-bisphosphate into the second messengers inositoltrisphosphate and DAG (Hashimotodani et al., 2007; Minichiello, 2009), and DAG is converted to the endogenous cannabinoid 2-AG by the enzyme DGL. In the present study, we found that inhibiting DGL blocked eCB-mediated iLTD, and exogenous BDNF-induced eCB release is also blocked by inhibiting DGL (Lemtiri-Chlieh and Levine, 2010). In addition, 2-AG has been shown to be the downstream effector of mGluR-dependent eCB release in many studies (for review, see Mackie, 2008; Katona and Freund, 2012; Ohno-Shosaku and Kano, 2014). However, a contributing role for anandamide cannot be ruled out, as several studies have shown a link between mGluR signaling and anandamide generation (Azad et al., 2004; Puente et al., 2011; Edwards et al., 2012; Huang and Woolley, 2012), presumably through PLC signaling (Liu et al., 2008).

In the current study, eCB-mediated iLTD was induced using TBS protocols that are similar to protocols that induce LTP at excitatory synapses, in which endogenous BDNF also plays a critical role (Aicardi et al., 2004; Abidin et al., 2006; Lu et al., 2010). For example, Lu and colleagues (2010) have shown that BDNF facilitates LTP by suppressing GABAergic inhibition and enhancing pyramidal neuron excitability. Thus, endogenous BDNF may enhance LTP and/or postsynaptic excitability at least in part by triggering eCB release which then induces long-term depression of inhibitory synapses, in addition to the direct effects of BDNF, as well as eCBs, at excitatory synapses. Interestingly, endogenous BDNF has also been shown to play a role in the induction of GABAergic LTP in the developing rat hippocampus (Gubellini et al., 2005). In order to fully understand the net effect of a given stimulation protocol on cortical circuits, it will be important to consider simultaneous changes at both excitatory and inhibitory synapses.

The present study focused on the role of BDNF in long-term eCB-mediated plasticity at inhibitory synapses. BDNF-induced endocannabinoid release may also play a role in eCB-dependent short-term synaptic plasticity at inhibitory synapses (DSI) or excitatory synapses (DSE). In fact, exogenous BDNF has been shown to enhance DSE via activation of the immediate-early gene *homer1a* (Roloff et al., 2010). It will therefore be interesting to explore whether endogenous BDNF contributes to eCB mobilization during DSI and DSE and whether it has synergistic effects with depolarization-induced eCB release.

There is increasing evidence for multiple types of interactions between BDNF and the eCB system. BDNF, for example, regulates CB1R expression as well as its sensitivity to endogenous agonists in cultured cerebellar granule neurons (CGNs) (Maison et al., 2009). De Chiara and colleagues (2010, 2013) found that in striatum, BDNF selectively antagonizes CB1R receptor function at inhibitory synapses in a cholesterol metabolism-mediated mechanism It has also been found that the neuroprotective effects of eCBs, particularly in depression and epileptic seizures, are mediated by changes in BDNF expression (Marsicano et al., 2003; Khaspekov et al., 2004; Aguado et al., 2007; Aso et al., 2008; Vinod et al., 2012). During interneuron differentiation, eCBs have additive effects with BDNF in regulating interneuron migration (Berghuis et al., 2005). A full understanding of the physiological roles of each of these systems will require a greater mechanistic understanding of their synaptic interactions. The current study, describing a novel form of eCB-mediated LTD at inhibitory synapses that requires endogenous BDNF/trk signaling, may contribute to this understanding.

**Table:** Statistical table

	Data structure	Type of test	Power
153	Normal distribution	Paired *t* test	0.97
169	Normal distribution	Paired *t* test	0.70
184	Normal distribution	Paired *t* test	1
190	Normal distribution	Paired *t* test	0.99
191	Normal distribution	Paired *t* test	0.99
236	Normal distribution	Paired *t* test	0.65

## Synthesis

The decision was a result of the Reviewing Editor Pablo Castillo and the peer reviewers coming together and discussing their recommendations until a consensus was reached. A fact-based synthesis statement explaining their decision and outlining what is needed to prepare a revision is listed below. The following reviewer agreed to reveal his identity: David Lovinger

The reviewers understand the evidence for a role of BDNF and eCBs in TBS-induced LTD is reasonably good in the study, but there are several concerns about the experiments examining pharmacological blockade of LTD, as well as the small sample sizes in some experiments.

Here is a list of experiments requested by the reviewers:

a) Amplitude of the synaptic responses. The baseline IPSC amplitudes are generally quite large (nA) and variable (given the 30-70% of maximum criterion), raising concerns about voltage- and space-clamp that might distort the apparent magnitude of plasticity. However, a larger concern is the smaller amplitude responses in some experiments in which LTD was blocked. This is especially evident in figure 4, where amplitudes are often below 1 nA, and LTD is observed in panel 4B in the one cell showing a larger amplitude IPSC. Even in figure 1B it appears that LTD is larger when the baseline IPSC amplitude is larger. It would be best to include additional controls with lower-amplitude IPSCs to be sure that LTD is robust under this condition.
b) Increase the number of experiments. The group sizes are sometimes as small as n = 3 or 4 (e.g. figures 3D, 4C). Sample sizes of at least 6 per group would be better.
c) Recording stability. Demonstrate stability of the recordings (i.e. ∼40 min recordings, no iLTD induction, no drugs in the bath). In addition, longer baseline recordings at least 10 min, are required. There is no need to duplicate the whole study.
d) CB1R blockade. Provide some justification for using AM251 in Fig. 1 and SR141716A in Fig. 5. Both data sets should be obtained in the presence of the same inhibitor at identical concentration.
e) Demonstrate that eCB-dependent iLTD is presynaptically expressed. At the very least, analyze paired-pulse ratio before and after iLTD induction.
f) Examine the role of 2-AG. The authors speculate that this is the endocannabinoid involved in this LTD, and this is a testable hypothesis
g) Use an alternative way to demonstrate the role of PLC in iLTD. The U-73122 drug has now been shown conclusively to lack any specificity for PLC. To quote from Huang et al., ACS Chemical Biology, 2011, "Several small molecule PLC inhibitors are indeed reported in the literature, among which U73122 is the most widely used. However, U73122 contains an electrophilic maleimide moiety that reacts with diverse nucleophiles including glutathione and various uncharacterized lipid components. More importantly, the direct inhibition of phospholipase activity by U73122 has not been demonstrated with purified PLCs. Instead, U73122 inhibits several other proteins including various calcium pumps, 16 phosphatidylinositol-4-phosphate kinase,17 and 5-lipoxygenase." The authors should either find a better way to try to implicate PLC in this LTD, or leave this out of the paper.
h) Positive control that NMDARs and group I mGluRs are effectively blocked during repetitive stimulation. CPP is a competitive antagonist that can be unblocked by repetitive stimulation. Test whether 3 uM CPP blocks NMDAR-EPSCs elicited by the same repetitive stimulation used to induce iLTD. Likewise, provide positive control that 500 uM E4CPG blocks group I mGluRs.
i) Test the role of postsynaptic calcium. The authors discuss the potential role of intracellular calcium in BDNF release, but never examine if the LTD they observe is dependent on postsynaptic calcium. While iGluRs and mGluRs may not be involved in this LTD, perhaps other calcium sources play a role, and this should be examined.
j) Double check that DMSO and BSA have no significant effect on synaptic transmission.
k) Revise analysis. Several experiments include comparisons of multiple groups, so analysis with ANOVA would be more appropriate than t-tests.

Text modifications:

– The evidence for presynaptic BDNF release is weak and should be toned down. It is unlikely that BDNF could be released from presynaptic GABAergic terminals as BDNF mRNA is not expressed in interneurons (see Ernfors et al, Neuron, 1990). Moreover, the authors cannot discard dendritic release of BDNF from neighboring neurons activated by the iLTD induction protocol (see Kuczewski et al., 2008). Besides, they must demonstrate that both NMDARs and mGluRs are fully blocked during iLTD induction.

– The authors may want to discuss the data from the Gaiarsa lab (see e.g. Gubellini et al.) regarding effects of endogenous BDNF on GABAergic transmission.

– Indicate the pre-incubation time of all drugs used in this study.

– In all cases where review articles are cited, identify these references as such; .e.g., "for a review, see xyz".
